# Phytoplankton chlorophyte structure as related to ENSO events in a saline lowland river (Salado River, Buenos Aires, Argentina)

**DOI:** 10.1002/ece3.983

**Published:** 2014-02-24

**Authors:** Lía C Solari, Néstor A Gabellone, María C Claps, María A Casco, Karina P Quaíni, Nancy C Neschuk

**Affiliations:** 1 Instituto de Limnología “Dr. Raúl A. Ringuelet”, CCT Conicet La PlataBoulevard 120 y 62, 1900, La Plata, Buenos Aires, Argentina; 2 División Ficología, Museo de Ciencias Naturales de La PlataPaseo del Bosque s/n, 1900, La Plata, Argentina; 3 Departamento de Estudios Ambientales de la Dirección Provincial de Saneamiento y Obras Hidráulicas del Ministerio de Infraestructura, Vivienda y Servicios Públicos de la Provincia de Buenos Aires7 N° 1267, 1900 La Plata, Buenos Aires, Argentina

**Keywords:** Argentina, chlorophytes, El Niño–La Niña–Southern Oscillation events, *Monoraphidium*, plankton, saline lowland river, the indicator value

## Abstract

We analyzed the phytoplankton present in the lower sector of the Salado River (Buenos Aires, Argentina) for 10 years (1995–2005) and detected significant changes occurring in chlorophyte abundance and species richness during La Niña event (1998–1999), which period was analyzed throughout the entire basin (main stream and tributaries). We compared the physicochemical and biologic variables between two El Niño–La Niña–Southern Oscillation (ENSO) periods – El Niño (March 1997–January 1998) and La Niña (May 1998–May 1999) – to identify possible indicators of a relationship between climatic anomalies and chlorophyte performance. Chlorophyte density increased during the La Niña. Under normal or extreme hydrologic conditions, mobile (*Chlamydomonas* spp.) and nonmobile (*Monoraphidium* spp.) chlorophytes codominated. These species belonged to Reynolds's functional groups X1 and X2, those typical of nutrient-enriched environments. Comparative analyses between El Niño and La Niña periods indicated significant differences in physicochemical (K^+^, dissolved polyphenols, particulate reactive phosphorus, alkalinity, pH) and biologic (species diversity and richness, phytoplankton and chlorophyte total densities) variables between the two periods at all basin sites. During the La Niña condition, species richness was greater owing to interconnected shallow lakes and drainage-channel inputs, while the Shannon diversity index was lower because of the high abundance values of *Monoraphidium minutum*. A detailed analysis of the chlorophytes in the entire basin, indicated that changes in density and species dominance occurred on a regional scale although diverse chlorophyte assemblages were identified in the different sectors of the Salado River basin. After La Niña event, the entire basin had the potential to revert to the previous density values, showing the resilience to global environmental changes and the ability to reestablish the general conditions of stability.

## Introduction

Streams and rivers in semiarid regions are sensitive to a changing climate because small climatic alterations can produce large hydrologic variations in those bodies of water. This amplified response can include the entire watershed (Dahm and Molles 1992). Hydrologic events caused by El Niño conditions have been clearly identified in rivers of South America (Mechoso and Perez-Iribarren 1992; Pisciottano et al. 1994; Caviedes and Waylen 1998; Kane 2000). These processes were particularly pronounced during the severe 1982–1983 and 1997–1998 episodes that influenced terrestrial ecosystems primarily by altering patterns of rainfall, surface temperature, and sunlight availability, thus affecting primary productivity, plant and animal mortality, and species-specific reproductive strategies (McPhaden et al. 2006). Nieto Ferreira et al. (2003) established that during 1998 and 1999 clear contrasting Southern Oscillation conditions alternated. In subtropical South America, where the Salado River basin is located, during the summer of 1998 high precipitations related to massive, long-lived convective cloud systems occurred (an El Niño event). Vadadi-Fülöp et al. (2012) recognized that in freshwater environments, changes related to floods and droughts can alter the abiotic variables as well as the biologic patterns. Environmental parameters controlling phytoplankton biomass in lowland rivers include flushing, surface and underwater light, temperature, sedimentation, and zooplankton grazing rate (Garnier et al. 1995). All these influences may become altered, either directly or indirectly (e.g., grazing) as a result of climate change (Johnson et al. 2009). Moreover, the consequences of human activities resulting in atmospheric alterations (e.g., greenhouse effects) could be adding onto the early stages of the El Niño–La Niña–Southern Oscillation (ENSO) phenomenon or even synergizing with it (McPhaden et al. 2006).

Phytoplankton variations have been found to be related to ENSO events in the Paraná River, where alterations in algal density, biovolume, and community structure were mainly associated with fluctuations in the flow discharge (Zalocar de Domitrovic et al. 2007; Devercelli 2010). Nevertheless, the causality of these phenomena has not yet been clarified. As El Niño and La Niña episodes produce changes in the proportion of species characteristic of normal hydrologic regime but do not result in a substitution of other species, the algal community can be thought to be rapidly restructured at the end of an event through the manifestation of an inherent structural resilience (Reynolds 1997). As variations in the water level allow an enrichment of river water with more abundant algal populations than those introduced previously, those environments can act as storage zones. The slow but progressive increase in algal concentration as the hydrometric level decreases accompanied by the corresponding fluxes in dominant taxonomic groups is compatible with the notion referred to by Huszar and Reynolds (1997) of a gradual hydroclimatic change, resulting from internal processes (Zalocar de Domitrovic et al. 2007). Increased carbon dioxide in the air has also been hypothesized as causing an enhancement in algal biomass and in the productivity of planktonic algae so as even to produce a bloom. Water alkalinity seems also to be related because of the possibility that algae could incorporate carbon in the form of bicarbonate as well as carbon dioxide from the air (Schippers et al. 2004).

In the Salado River basin (Buenos Aires, Argentina), long-term studies were performed from 1995 through 2005. These investigations were initially carried out on the lower basin (Neschuk et al. 2000, 2002a,2002b; Gabellone et al. 2001; Solari et al.^2002^) and subsequently on the entire watershed and main tributaries (Gabellone et al. 2008, 2010). The Salado River exhibits wet and dry periods (Moncaut 2003), intense land use at the headwaters (Gabellone et al. 2005), and a fluctuating salinity concentration resulting from hydrologic changes that affect zooplankton structures over space and time (Claps et al. 2009).

We consider that (1) the phytoplankton's biodiversity is conducive to a resilience to changes occurring in the basin, but not to the global environmental alterations. (2) The notable changes in the abundance of dominant phytoplankton species related to post-Niño period (La Niña event) are not restricted to the lower river sector, but rather occurred throughout the entire basin. (3) The phytoplankton maintain their structure over the whole basin, including the tributaries, in spite of the hydrologic fluctuations – with the sole exception of the chlorophytes, and especially *Monoraphidium minutum*. The main objectives of this investigation were to characterize the alterations in the phytoplankton structure resulting from the environmental changes ensuing over a period of 10 years in this lowland river; to compare the phytoplankton structure along with the physicochemical parameters occurring between El Niño and La Niña phases during that time; and to analyze the phytoplankton assemblages that characterized the entire basin including the main channel and natural and artificial tributaries during La Niña condition of that period.

## Materials and Methods

### Study area

**Figure 1:**
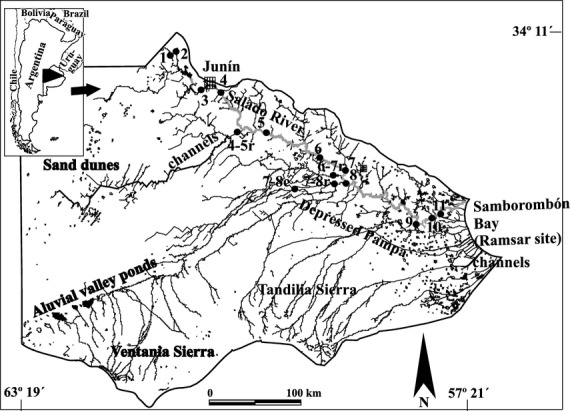
Sampling sites in the Salado River basin: St. 1, Salado Stream; St. 3, Junín 1; St. 4, Junín 2; St. 4–5r, Saladillo stream; St. 5, Achupallas; St. 6, Ruta 30; St. 6–7r, Saladillo–Vallimanca Stream; St. 7, Roque Pérez; St. 7–8c, Canal 16; St. 7–8r, Las Flores Stream; St. 9, General Belgrano; St. 10, El Destino; St. 11, La Postrera.

The Salado (Fig. 1) – a typical lowland river and the major autochthonous one of the Buenos Aires province – is the southernmost tributary of the Río de la Plata basin, with a watershed of around 150,000 km^2^. The river has a length of approximately of 571 km and a low slope (mean, 0.107 m·km^−1^) with a flow rate reaching no more than 100 m^3^·sec^−1^ in dry periods but increasing to up to as much 1500 m^3^·sec^−1^ during floodings, with consequent variations in conductivity and the transport of dissolved and particulate materials. The basin includes one of the most productive agricultural regions in the country (Gabellone et al. 2005). The regime of the Salado River is quite variable, and unfortunately, not all the hydrologic information about the sites and sampling periods of this study could be obtained. A complete description of Salado River basin, however, is found in Gabellone et al. (2005).

**Figure 2:**
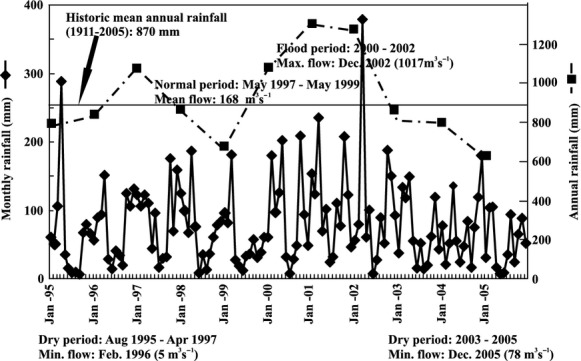
Annual and monthly rainfall of the Salado River basin during the sampling period (1995–2005), with the main hydrologic cycles identified in the basin.

The period analyzed (1995–2005) throughout the entire basin contained marked meteorologic and hydrologic changes. The mean annual rainfall (924 mm) for the study period was slightly higher than the historical mean of the basin for the years 1911–2005. In the beginning of 1995 and 1996, the quantity of rainfall was similar to the mean. In 1997, coinciding with the beginning of an El Niño condition, the annual rainfall was 1057 mm. During the next 2 years, a decrease in precipitation was observed (for 1998 and 1999, 749 mm, characterizing a La Niña event). From 2000 to 2002, a flooding period occurred because of maximal rainfalls. During 2003–2005, the rainfall decreased with the lowest value (626 mm) being recorded in 2005. At La Postrera (St. 11), the discharge (400 m^3^·sec^−1^) was slightly higher than the mean value estimated for this sampling station (300 m^3^·sec^−1^) at the beginning of the study period (May 1995) related to the quantity of rainfall recorded during the previous month (239 mm; Fig. 2). Later in the same year and during the next two, the discharge values were low – for example, for the summer of 1996 a minimum occurred at 5 m^3^·sec^−1^. During 1997, in spite of the extent of the rainfall recorded, the discharge was not high as the rainfall occurred mainly in summer, a season characterized by a maximum level of evaporation. During the winter of 1998–1999, the discharge oscillated at around the mean value estimated for this site. Finally, coinciding with a flooding period, the maximum discharge was recorded in December 2002, but over the next 3 years, a decrease in discharge took place because of the low levels of rainfall that subsequently occurred (December 2005; Figs. 2, 4).

### Field sampling and laboratory methods

The information thus obtained enabled the development of a series of indices related to ENSO, such as the multivariate index ENSO among others (Wolter and Timlin 1993). The ENSO multivariate ENSO index (MEI) values corresponding to the present sampling occasions were downloaded from http://www.esrl.noaa.gov/psd/enso, while the monthly mean temperature values compared with ENSO variations were obtained from the meteorological station nearest to the lower Salado River basin (and St. 11) corresponding to the city of Dolores. The information on the average monthly insolation incident on a horizontal surface was downloaded from the NASA official site (https://eosweb.larc.nasa.gov/).

Seasonal samples were obtained during the period March 1997–May 1999 in thirteen locations; with the sites sampled covering the headwaters, the middle and lower sectors of the main channel, and the main tributaries. During the period 1995–2005, a total of 31 mainly seasonal samples were obtained at one site (St. 11, at La Postrera, some 84 km from the mouth; Fig. 1; Neschuk et al.2002a, 2002b; Solari et al. 2002). The 1995–2005 data have been included for the purpose of comparison with the corresponding figures obtained during the 1997–1999 period. The sampling stations (Fig. 1) had already been established and cited in Gabellone et al. (2005).

The sites were visited initially in March 1997 and then quarterly on a seasonal basis up to the autumn of 1999. The period was divided into two parts: in the first (March 1997–January 1998), the main occurrence was an El Niño event, in contrast to the second (May 1998–May 1999) where the principal feature was a La Niña condition.

The water temperature, conductivity, pH, dissolved oxygen concentration, and turbidity were measured in situ with a Horiba Multimeter U10. Water samples were collected for the following chemical analyses through the use of the following techniques from APHA (1995). The total phosphorus, dissolved reactive phosphorus, and particulate reactive phosphorus were determined by the ascorbic acid method after digestion with acidic persulfate (method 4500-PB). Dissolved polyphenols and chloride were determined according to the methods 5550 B and 4500-Cl B, respectively. The sodium and potassium concentrations were measured by flame photometry (methods 3500 Na D and 3500 K D, respectively). The concentrations of total suspended solids were measured according to method 2540 D, and the total alkalinity determined by titration (method 2320 B). The concentrations of particulate organic matter were measured by loss on heating at 550°C (method 2540 E), and dissolved organic carbon estimated indirectly in water filtered through Whatman GF/F filters by the optical density at 440 nm (Kirk 1983).

Phytoplankton samples were obtained with a 2-L Van-Dorn bottle and preserved with 1% (w/v) acidified Lugol's iodine solution. Counts were made after Utermöhl (1958) with an inverted microscope. Chambers of 5 or 10 mL were used depending on phytoplankton density. A maximum counting error of 20% was considered acceptable in estimating the abundance of each major algal group.

The *M. minutum* biovolume (mm^3^·L^−1^) was calculated by applying the measurements of dimensions to the appropriate formula for a corresponding geometrical figure (Hillebrand et al. 1999; Sun and Liu 2003). The calculations in terms of carbon biomass were made by the Menden-Deur and Lessard (2000) formula.

### Statistical analysis

Seventy-two species at densities above 500 ind. mL^−1^ on each sampling event (during the period 1997–1999) and present on at least three sampling occasions were selected from a total 97 chlorophytes. The criteria for species selection were general enough to include rare, uncommon, or occasional species that at some sampling manifested a sufficient density to be considered a population.

To analyze the differences in principal chemical and biologic variables in the 1997–1998 and 1998–1999 periods, the Student's *t-*test was used. A nonhierarchical k-means clustering was applied to preselected clusters of the different species and the various sites (Dufrêne and Legendre 1997). The indicator value (IndVal) was used to identify indicator species in each group once the grouping of the sites was determined on the basis of the species assemblage and the species obtained at each site (by the k-means procedure after Dufrêne and Legendre 1997). According to the occurrence and abundance of the chlorophytes, the analyses performed allowed a classification of different sampling stations within the basin. The species present in three or more groups with IndVals higher than 10 were considered as all habitat.

The species diversity of the chlorophytes was estimated by means of the Shannon–Weaver index (Ludwig and Reynolds 1988).

## Results

### Period from May 1995 through February 2005

**Figure 3:**
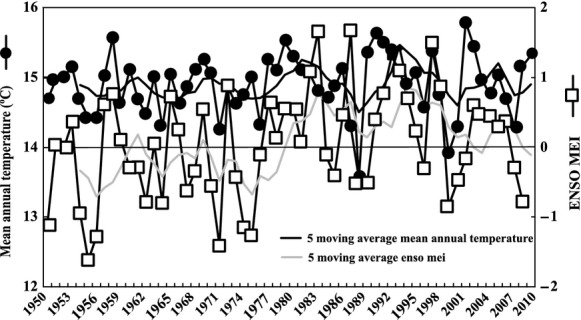
Oscillations in the mean annual temperature and multivariate ENSO index (ENSO-MEI) values during the 1950–2010 period in the lower basin of the Salado River (St. 11).

Two significant climatic changes with respect to ENSO events occurred during the study period (1995–2005): an El Niño (1997–1998) and a La Niña (1998–1999). These oscillations caused marked changes in the mean air temperature of the lower Salado River basin (Fig. 2), but the average insolation did not differ between the two periods (El Niño 4.62 and La Niña 4.63 kWh·m^−2^·day^−1^). The average air temperature was higher during El Niño (15.4°C) than during La Niña (13.9°C) event. Since 1982, the tendency lines of the moving averages of the mean annual temperature and the ENSO-MEI exhibited the same pattern, one that contrasted with those observed from 1950 to 1980 (Fig. 3).

**Figure 4:**
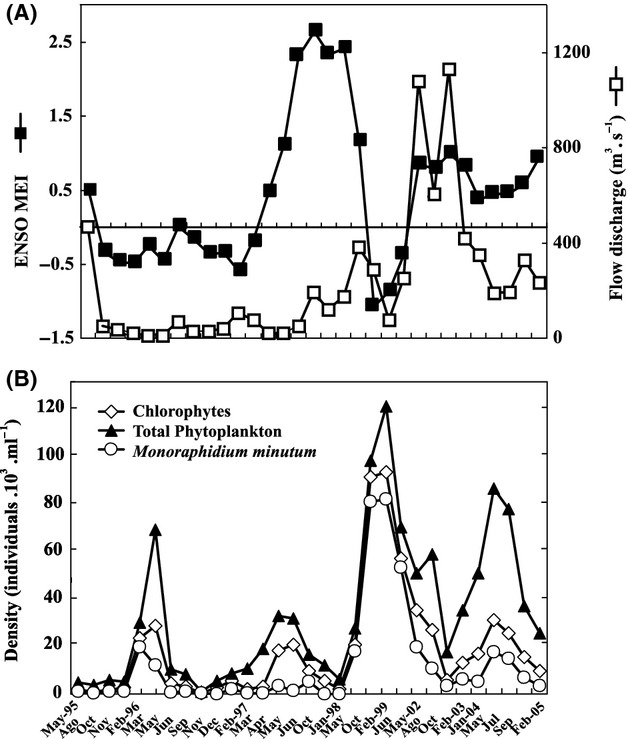
The flow discharge and the multivariate ENSO index (ENSO-MEI) values (A) and variation in the density of the total phytoplankton, total chlorophytes, and *Monoraphidium minutum* (B) during the sampling period.

The chlorophyte density increased markedly – by around one order of magnitude – during La Niña condition (Fig. 4) with a notable increase in total phytoplankton density. Throughout the entire 1995–2005 period in the lower sector of the Salado River, chlorophytes (species of *Crucigenia*, *Monoraphidium* and *Chlamydomonas*) were dominant in some fashion in the Salado River, with those predominating in 67% of the samples and being codominant in the rest. The mean value for chlorophyte participation within the total phytoplankton abundance was 49%, whereas the cyanobacteria represented 27%, the diatoms 20%, the pyrrophytes 2%, and the euglenophytes 2%.

### Period from March 1997 through May 1999

Likewise, during the period 1997–1999, the chlorophytes had been dominant throughout the entire basin, including main channel and tributaries (in 80 of 104 samples).

The results of comparative analyses between El Niño and La Niña periods indicated that certain physicochemical variables (e.g., K^+^ concentration, dissolved polyphenols, particulate reactive phosphorus, alkalinity, and pH) were significantly different between the two events at two sites in the lower basin (sts. 10 and 11). The diversity index and richness along with the total phytoplankton and total chlorophyte densities furthermore differed significantly (*t-*test at a *P* < 0.05; Table 1). The density of *M. minutum* was two orders of magnitude higher than that of the other species, thus making a significant contribution to the differences between the total phytoplankton and the total chlorophyte densities. The total phytoplankton density and the chlorophyte species richness were lower during El Niño than La Niña condition; but, in contrast, the total phytoplankton and the chlorophyte Shannon–Weaver indices were higher during El Niño than La Niña period (Table 1).

**Table 1:** Mean values of the variables for the two periods analyzed (El-Niño: Mar 1997–Jan 1998, and La Niña May 1998–May 1999) in stations 10 and 11.

	El Niño	La Niña	*t*-Test
Conductivity (*μ*S·cm^−1^)	4357 (1990–6260)	3373 (1130–7900)	ns
K^+^ (mg·L^−1^)	36 (18–54)	22 (13–35)	^**^
Na^+^ (mg·L^−1^)	667 (539–885)	481 (144–1173)	ns
Cl^−^ (mg·L^−1^)	979 (329–1470)	767 (414–1805)	ns
Na^+^/K^+^	34 (23–21)	39 (15–53)	ns
Alkalinity (mg·L^−1^)	352 (254–472)	425 (218–628)	^*^
pH	8.7 (8.3–9.2)	8.1 (7.6–9.2)	^*^
Total phosphorus (*μ*g·L^−1^)	354 (130–546)	304 (120–431)	ns
Particulate reactive phosphorus (*μ*g·L^−1^)	29 (9–81)	70 (24–158)	^*^
Dissolved reactive phosphorus (*μ*g·L^−1^)	120 (59–214)	95 (58–120)	ns
Particulate organic matter (g·L^−1^)	0.031 (0.001–0.066)	0.030 (0.003–0.027)	ns
Optical density 440	0.085 (0.055–0.135)	0.064 (0.035–0.120)	ns
Dissolved polyphenols (mg·L^−1^)	1.55 (1.00–2.24)	2.26 (1.66–2.99)	^**^
Suspended solids (g·L^−1^)	0.127 (0.011–0.205)	0.083 (0.027–0.206)	ns
Temperature (°C)	17.6 (6.2–24.6)	17.3 (12.6–21.4)	ns
Phytoplankton density (individuals·mL^−1^)	14812 (2567–39734)	97589 (69578–234660)	^**^
Chlorophytes density (individuals·mL^−1^)	4684 (1024–10468)	65900 (20242–99337)	^**^
Chlorophytes richness	14 (6–23)	24 (11–38)	^*^
*Monoraphidium minutum* density (individuals·mL^−1^)	558 (0–3315)	57327 (17654–84920)	^**^
*M. minutum* biomass (*μ*g C·L^−1^)	1.48 (0–7.87)	114.45 (37.89–165.52)	^**^
Chlorophytes diversity	2.19 (1.73–2.69)	0.68 (0.45–1.01)	^**^
Phytoplankton richness	29 (17–41)	49 (25–74)	^*^
Phytoplankton diversity	2.0 (0.39–2.87)	1.40 (0.77–2.12)	^*^
Zooplankton density (individuals·L^−1^)	456 (184–740)	653 (213–1670)	ns

The comparison was made with the *t-*test (minimum and maximum values in brackets, ^*^P < 0.05, ^*^^*^P < 0.01, *n*: 8).

During El Niño event, the biovolume of *M. minutum* was 177 10^3^ *μ*m^3^ with a carbon production of 18 *μ*g C·L^−1^, whereas these respective maxima rose to 1.947 10^3^ *μ*m^3^ and 174 *μ*g C·L^−1^ during the subsequent La Niña condition.

### Period from May 1998 through May 1999 (La Niña)

During this period, the Salado River basin (13 sampling stations) was characterized by alkaline (mean pH: 8.6) and oxygenated (mean dissolved oxygen concentration: 7.7 mg·L^−1^) water and a relatively high water temperature (mean: 18.7°C). The mean conductivity was 3373 *μ*S·cm^−1^ (1130–7900 *μ*S·cm^−1^) throughout.

The Salado River's phytoplankton was dominated by chlorophytes (86 Chlorococcales, seven Zygnematales, two Volvocales, and two Ulotrichales (Table S1). The chlorophytes were dominant in 96% of the samples (*n* = 52).

**Figure 5:**
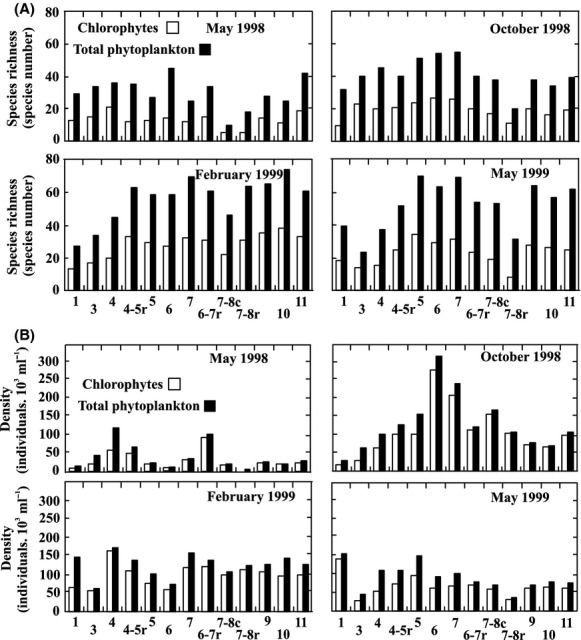
Variation in (A) the species richness of total phytoplankton and chlorophytes and (B) the density of total phytoplankton and chlorophytes during the La Niña period (May 1998–May 1999).

The mean percent richness of chlorophytes with respect to the total phytoplankton richness in the Salado River basin (Fig. 5A) was similar on all sampling occasions (mean, 46%; SD, 7; *n* = 52). The minimum species richness of chlorophytes occurred in the tributary sites (sts. 7–8c, and 7–8r, with five species at each site in May 1998). The maximum mean chlorophyte species richness was registered in February 1999 (at 28 species), and the highest value was recorded at two sites in the lower river sector (St. 10: 38 species and St. 9: 35 species).

The maximum species richness per station (Fig. 5A) occurred in the middle basin (sts. 4–5r and 6–7r) and lower basin (sts. 9–11).

The chlorophytes were the most abundant phytoplankton group in the river (at a mean relative abundance of 76%). The minimum absolute abundance was registered in May 1998 during a diatom dominance, while the maximum densities occurred in October 1998 at two sites (St. 6 and St. 7) owing to the contribution of the Volvocales (Fig. 5B). The chlorophyte density in the main channel and tributaries was more uniform in February 1999, but minimum mean densities occurred in May of 1998 (mean 39.3 10^3^ individuals·ml^−1^) and maximum in October 1998 (10.7 10^4^ individuals·ml^−1^). The maximum density per station was registered in the middle sector (sts. 6, 7, and 4–5r) but diminished in the lower sector (sts. 9 and 10) except at St. 11, where the density was similar to that of the middle sector because of the phytoplankton contribution from the shallow lakes associated with the river at that point. The mean minimum density per station was registered at St. 3.

**Figure 6:**
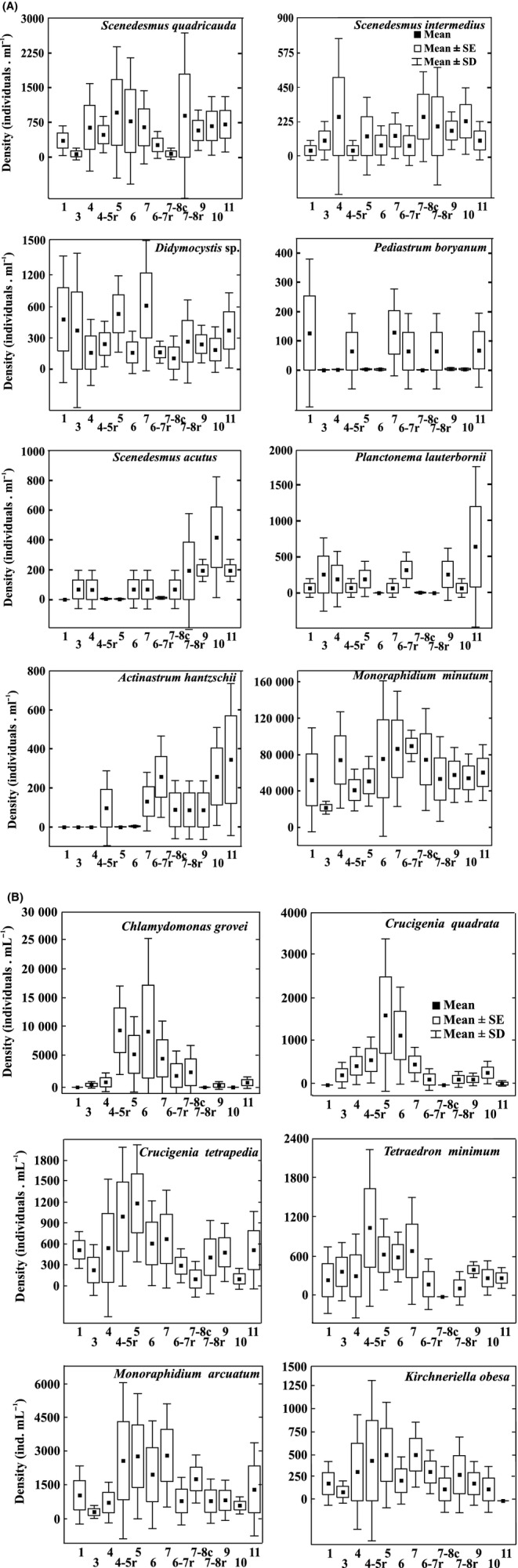
(A) and (B) Density of species characteristic of the main channel, tributaries, and lower basin of the Salado River during the sampling period (March 1997–May 1999).

*Monoraphidium minutum*, the most conspicuous chlorophyte species, exhibited a maximum abundance in the middle sector, while species of *Chlamydomonas* were codominant (Fig. 6A and B). The spatial distribution with respect to site of the mean densities of mainly chlorophyte species was variable in the basin. *M. minutum* reached a maximum mean density in the middle basin with a wide dispersion over the different sampling months because of particularly low values in May of 1998. In the lower basin, however, this species registered lower mean densities but with less standard deviation and a greater degree of homogeneity throughout that portion of the river. The minimum densities of *M. minutum* occurred at St. 3, while at the same time, the low densities of that species at St. 4–5r failed to affect the chlorophyte density in the river's main channel because *Chlamydomonas grovei*, *Crucigenia quadrata*, *Crucigenia tetrapedia, Kirchneriella obesa, Monoraphidium arcuatum*, and *Tetraedron minimum* exhibited high densities in this same tributary that, in turn, influenced the middle sector of the river. These species also showed a clear spatial distribution of unimodal abundance along with a marked decrease in density within the lower basin (Fig. 6B). Species of *Scenedesmus* (*S. intermedius, S. quadricauda*) and *Didymocystis* sp. had high densities throughout the main channel of the river. *Actinastrum hantzschii*, *Planktonema lauterbornii*, and *Scenedesmus acutus* were abundant in the lower basin, while *Pediastrum boryanum* was represented prominently in the tributaries (Fig. 6A).

### Multivariate analysis

#### Nonhierarchical analysis, k-means clustering

##### Sites

**Figure 7:**
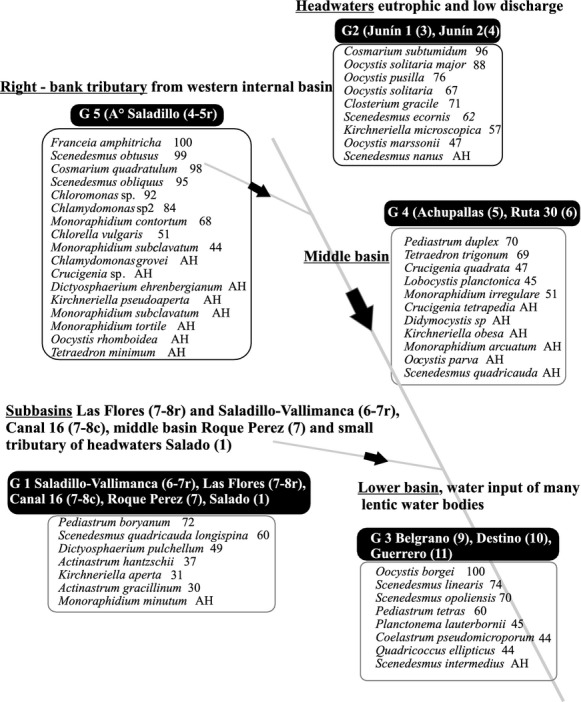
Indicator value of most abundant species of chlorophytes after assemblage of the species into groups obtained by k-means clustering during the sampling period (March 1997–May 1999). AH, all-habitat species.

Group 1 consisted of sites on the tributaries of the right margin of the river (sts. 7–8r and 7–8c), the first tributary of the headwaters (St 1), St. 7 on the main river course, and St. 6–7r near to the mouth of Saladillo–Vallimanca Stream. Group 2 comprised two sites at the headwaters (sts 3 and 4). In this sector, the river received a contribution from the shallow lakes situated in the upper basin (the Gómez, Mar Chiquita, and El Carpincho shallow lakes). This portion of the river was characterized by a low mean flow from the tributaries of the right bank confluence. Group 3 consisted in the three sites in the lower basin (sts 9–11). In this sector, the river is connected either directly or indirectly to many shallow lakes, backwater ponds, and flushing lakes on both the right and left margins. Group 4 was composed of the stations located at the middle sector of the main channel (sts. 5 and 6), while Group 5 contained only the single site St. 4–5r on the Saladillo Stream. Before flowing into Salado River, the Saladillo Stream drains water from the western inner basin that passes through canalizations and lake systems. The conductivity of the Saladillo Stream is usually higher than that of the river and influences the two sites located immediately downstream (sts. 5 and 6) to an extent depending on the variation in the flow (Fig. 7).

##### Species

Group 1 contained species valued at a high distance from the center of the cluster: *Dictyosphaerium pulchellum, Kirchneriella aperta*, and *M. minutum* were frequent at all the river sites without becoming identified with any particular one. Nevertheless, *P. boryanum* was present in locations such as at the tributary of the headwaters (St. 1) and within the middle sector of the river (St. 7). Group 2 was fairly well consolidated and included few species either near or at a distance from the center of the cluster. The following constituent species were well representative of sts. 3 and 4: *Cosmarium subtumidum, Oocystis marssonii, Oocystis pusilla, Oocystis solitaria*, and *Oocystis solitaria var. major*. Group 3 contained species characteristic of the lower basin (sts. 9–11). *Oocystis borgei* was present in only this sector of the river (sts. 9–11), while *Monoraphidium irregulare, Scenedesmus acuminatus,* and *S. opoliensis* had their maximum densities in this zone as well. Group 4 consisted of species registered in two stations of the middle sector of the river (sts. 5 and 6). In addition, species of the genera *Crucigenia, Diplochloris* sp., *Tetraedron minimum*, and *Lobocystis planctonica* were registered at a maximum abundance in this sector of the river. Group 5 comprised species clearly consolidated. All of these species were typically present in the Saladillo Stream. Moreover, *Monoraphidium contortum,* and *Dictyosphaerium ehrenbergianum* were also present in this stream and rose to high densities in the main channel of the Salado River downstream from the confluence with the Saladillo Stream.

##### Indicator Value

The spatial distribution of certain species of chlorophytes was not homogeneous throughout the basin, with certain members being clearly closely associated with different sectors of the river or its tributaries (Fig. 7). The sites differing most markedly were in the upper basin (sts. 1, 3, 4, 5), the middle basin (sts. 6, 7) – that latter portion being influenced by the contribution of the Saladillo Stream (St. 4–5r) – and the tributaries (sts. 7–8r and 7–8c) along with the lower basin, it being affected by the Saladillo–Vallimanca Stream (St. 6–7r). *Monoraphidium minutum* was an all-habitat species and accordingly exhibited a uniform spatial distribution (Fig. 7). Of the species that characterized the different tributaries, certain ones inhabited the river, but others were present in only the tributaries. Some were registered in only the Saladillo Stream (Group 5). These species are not representative of the groups obtained by the multivariate analysis mainly because of their low density. Group 2 that included sts. 3 and 4 also contained high-fidelity species (Fig. 7). In certain groups, such as Group 1, several species had a low IndVal either owing to a stenoic characteristic or because they were assessed as all habitat. On the other extreme, Group 5 contained numerous species with elevated IndVals because of their fidelity to specific environmental characteristics. In the headwaters (groups 2 and 5), the species present were registered with higher IndVals, whereas in the main channel downstream from the middle basin, the IndVals diminished (Fig. 7).

## Discussion

The phytoplankton of the Salado River was characterized by a high abundance and species richness of the chlorophytes, and not by a prevalence of diatoms, as is usual in the lowland rivers of temperate regions (Gosselain et al. 1994; Garnier et al. 1995; Lair et al. 1998; Train and Cleide Rodrigues 1998; Ietswaart et al. 1999; Leland et al. 2001; Salmaso and Braioni 2008; Istvánovics et al. 2010).

During the study period (1995–2005), the structure of the phytoplankton in the Salado River exhibited no marked changes: the chlorophytes always dominated – whether high, low, or normal hydrologic conditions prevailed – unlike what occurred in the Paraná River. The chlorophyte structure was maintained without replacement of the characteristic members although the environment underwent hydroclimatic changes produced by El Niño–La Niña oscillation. This result is in keeping with the proposed by Reynolds (1997).

The only previous record in the Salado River was partial and corresponded to samples taken 10 years earlier than the present study (O′Farrell 1993). The samples were obtained between November 1987 and February 1989. The first two samplings coincided with the end of a marked El Niño event (1987–1988) and the last three with a La Niña period. The results obtained in the lower river sector in February 1989 are the only example of a situation similar to the one that occurred in the period 1998–1999, characterized by a codominance of Cyanophyta and Chlorophyta with an increase in the abundance and percent participation of chlorophytes.

An effect of El Niño condition 1997–1998 on the phytoplankton was also observed in the Paraná River (Zalocar de Domitrovic et al. 2007), but not as markedly as in the Salado. In both rivers, the phytoplankton – and specifically the Chlorococcales – achieved a higher density during the subsequent La Niña event than at this time (García de Emiliani and Devercelli 2003). In the Salado River, the increased chlorophyte density during that La Niña period was also accompanied by a higher average alkalinity groundwater contribution than during El Niño condition. In the Paraná River during the earlier El Niño event, the phytoplankton showed significant differences in species richness, diversity, and chlorophyte abundance compared with La Niña period (Zalocar de Domitrovic et al. 2007). In the Salado River during El Niño condition, the species richness was notably lower than during to La Niña event (41 species as opposed to 74) with certain species manifesting a sporadic presence. This difference probably resulted from contributions received by the main course of the Salado River from interconnected shallow lakes, backwater ponds, and a system of drainage channels emptying into the river at low water levels (Reynolds 2000; Gabellone et al. 2001; Solari et al. 2002). Upon comparing the environmental conditions (e.g., river discharge, connectivity, and mainstream-floodplain interactions) and fundamental phytoplankton characteristics during periods involving three different hydrometric levels, Devercelli (2010) – working in the Paraná River – found marked differences in density, biomass, and phytoplankton structure. In the Salado River, however, El Niño event did not produce high water levels as had been detected in the Paraná. The following year (1998–1999) constituted a dry period with a marked increase in the chlorophyte density. The previous year to El Niño (1996–1997) – also a low water period – was not characterized by the same increase in the chlorophyte abundance, with a codominance of diatoms and chlorophytes (Solari et al. 2002).

The species of *Monoraphidium* were adapted to the lotic conditions by their morphologic characteristics and life strategies (Reynolds and Descy 1996). This genus has been characteristically predominant in lowland rivers; and its species have been numerically dominant, a scenario likewise typical of several European rivers – for example the Danube (Schmidt 1994; Stoyneva 1994), the Meuse (Descy 1987; Gosselain et al. 1994), and the Seine (Garnier et al. 1995). *Crucigenia quadrata, M. minutum*, and *Tetraedron minimun* manifested the largest increases in density and were identified as species that preferred the summer season (Descy 1987; Bazzuri et al. 2010). The significant increase in nonmobile (*M. minutum*) and mobile (*Chlamydomonas* spp.) forms – more characteristic of the midstream sector of the Salado – was related to La Niña period. The two species had different strategies as well as representing differing functional groups, with *M. minutum* belonging to X1 and *Chlamydomonas grovei* to X2 (Reynolds 1997; Reynolds et al. 2002). These two groups are known to be well adapted to living in mixed environments and are in addition opportunistic species of small size and rapid reproduction (Devercelli and O‘Farrell 2013). In the Paraná River, the presence of species of functional groups X1 (*Monoraphidium* spp.) and J (species of *Pediastrum* and *Scenedesmus*) and an increase in functional Group F (species of *Oocystis*, *Kirchneriella*, and *Dictyosphaerium*) were observed during La Niña event in contrast to El Niño condition (Devercelli 2010). In the Salado River, the species mentioned by Devercelli (2010) – species of *Monoraphidium, Pediastrum, Scenedesmus, Oocystis*, and *Kirchneriella* – were always present and would thus appear to be associated with environments having limitless nutrients (Reynolds 1997), such as characterizes the Salado River.

The altered environmental conditions that resulted from this El Niño–La Niña oscillation influenced global patterns of primary production – that is, the fixation of carbon by plants – with domino effects that ascended to higher levels of the food chain in ecosystems both marine and terrestrial (Behrenfeld et al. 2001). In view of the increase in the density of *M. minutum* alone during La Niña condition in the present study, the contribution of carbon was one order of magnitude higher than during El Niño period. Phytoplankton furthermore can strongly influence climatic processes and biogeochemical rhythms, particularly the carbon cycle (Boyce et al. 2010).

In a study of phytoplankton blooms in a eutrophic flushing lake at the mouth of a river, Phlips et al. (2007) found a relationship between wet cycles (i.e., an El Niño event) with higher water turnover rates and a decline in blooms and dry periods (i.e., La Niña conditions), where the phytoplankton achieved a high density. If we compare their results for the period of 1997–1999, the phytoplankton dynamic was similar to that observed in the Salado River, but with cyanophytes as the dominant algal group in that study. The present results necessarily highlight the scenario that during the drier periods in the Salado River the lack of large zooplankton herbivores (Claps et al. 2009) result in a low grazing pressure on the chlorophytes, thus allowing their extensive growth (Lair et al. 1998).

During the 10-year sampling period (1995–2005), the increase in the density of the phytoplankton of several orders of magnitude – and mainly the chlorophyte *M. minutum* – that occurred in the lower Salado basin during La Niña event (1998–1999) after a marked El Niño period (1997–1998) was not a local phenomenon, but rather occurred throughout the entire 150,000 km^2^ of the watershed. Although the physicochemical parameters did not vary significantly among the sampling sites in the basin, *M. minutum* nevertheless exhibited elevated densities in the stations within main channel and in the major tributaries that were characterized by different ionic conditions, nutrient concentrations, and associations with other algae. *Monoraphidium minutum* is considered a species well acclimated to lotic environments for both its ability to adapt to nutrient pulses and its wide tolerance to differing salinities (Solari et al. 2002; Carrillo et al. 2009). This latter feature can be considered a distinct advantage in the Salado River as marked changes occur in the flow that produce dry and wet periods and result in notable changes in conductivities within the basin. Thus, the wide limits of tolerance to changes in salinity exhibited by *M. minutum* represent a fundamental characteristic enabling competition with other algal species for survival. These features furthermore enable *M. minutum* to adapt to environmental alterations that occur over short time intervals in a rapidly changeable habitat such as the Salado River.

In conclusion, a detailed analysis of the chlorophyte species in the basin enabled the recognition that changes in the density and dominant species occurred on a regional scale although the different subbasins showed peculiarities in terms of algal density, species richness, and the nature of the species present.

The main differences in chlorophyte abundance – and mainly that of *M. minutum* – between January 1998 (low density) and February 1999 (maximum density) were coincident with the anomalies observed by Nieto Ferreira et al. (2003) in the region as a consequence of ENSO. Although species diversity decreased during La Niña condition (though the index applied gives more weight to the equitability), the composition of the different associations of species did not differ markedly enough to indicate a significant change in the phytoplankton structure.

*Monoraphidium minutum* grew and dominated simultaneously in different algal assemblages throughout the basin. These assemblages did not change in their species composition or their constituent proportions during *M. minutum* proliferation. This constancy could indicate that the plankton community is resilient. Moreover, zooplankton density exhibited no significant differences between the two periods (1997–1998 vs. 1998–1999), even though a predominance occurred of small herbivores with low grazing efficiency that would consider *M. minutum* as palatable. Changes in trophic relationships between biologic compartments are usually weak or infrequent. If such changes occur, a genetic alteration in the species has resulted entailing a modification in adaptability so as to expand the organism's niche. During the study period, sexual reproduction was undetected, although with the methodology used that form of replication would be difficult to observe. The increase in abundance by two orders of magnitude indicated that the phytoplankton had previously not achieved its maximum density. That increase could be related to a sequence that involves an exceptional El Niño–La Niña oscillation, resulting in two marked cloud-associated anomalies (Nieto Ferreira et al. 2003). El Niño condition influenced algal growth negatively, which parameter later exhibited a form of positive-feedback response to La Niña event. The chlorophytes, mainly *M. minutum*, might possess greater adaptability in order to withstand adverse periods in the river during El Niño occurrence and thus be able to take advantage of the next favorable period. Phlips et al. (2007) found similar patterns for the same oscillation but involving cyanobacteria in a more eutrophic river of Florida (USA). According to those authors, a low water turnover favors cyanobacterial blooms. By contrast, in the Salado River, the chlorophytes that prefer high turbulence prevailed (functional group X1). Nevertheless, synergistic effects of hydrologic and meteorologic occurrences (e.g.*,* clouds) that promoted conditions for those algal abundance peaks cannot be ignored. In our example, even though the analysis involved a regional scale with different algal assemblages and ionic strengths in the water, the dominance of *M. minutum* that occurred was analogous to what would be expected to take place in a lentic circumstance with minimal heterogeneity. The meteorologic phenomenon remained for 6 months between October 1998 and February 1999 and affected a coccal species with a high reproduction rate that maintained cohorts having many individuals. After that La Niña event, the entire basin had the potential to revert to the previous density values, thus demonstrating a high degree of resilience to global environmental changes and the ability to reestablish the general conditions of stability as defined by Mc Cann (2000).

## Supporting Information

Table S1 Phytoplankton chlorophytes found in the Salado River (97 species) during the period 1998–1999.
