# Supplementary material for: Phytoplankton chlorophyte structure as related to ENSO events in a saline lowland river (Salado River, Buenos Aires, Argentina)
Source: Ecol Evol. 2014 Feb 24;4(7):918–32. doi: 10.1002/ece3.983 (PMC3997310; doi:10.1002/ece3.983)
Supplement: Table S1 — Phytoplankton chlorophytes found in the Salado River (97 species) during the period 1998–1999. [file ece30004-0918-sd1.doc]

Table S1. Phytoplankton chlorophytes found in the Salado River (97 species) during the period 1998-1999.

| **Chlorococcales** | *Nephrocytium agardhianum* Näg. |
| --- | --- |
| *Acanthosphaera* sp. | *Oocystis borgei* J. Snow |
| *Actinastrum gracillimum* G. M. Smith | *O. marssonii* Lemm. |
| *A. hantzschii* Lag. | *O. parva* W. West & G. S. West |
| *Ankyra judayi* (G.M. Smith) Fott. | *O. pusilla* Hansg. |
| *Ankistrodesmus* sp. | *O.rhomboidea* Fott |
| *Chlorella vulgaris* Beijerinck | *O. solitaria* Wittrock |
| *Chloromonas* sp. | *O. solitaria* var*. major* Wille |
| *Coelastrum astroideum* De Not. | *O. tainoensis* Kom. |
| *C. microporum* Näg. | *Oocystis* sp. |
| *C.pseudomicroporum* Kors. | *Pediastrum boryanum* (Turpin) Meneghini |
| *Coenochloris helvetica* Hind. | *P. boryanum* var *brevicorne*  A. Braun |
| *C. planconvexa* Hind. | *P. boryanum* var. *longicorne* Reinsch |
| *Crucigenia quadrata* Morren | *P. duplex* Meyen |
| *C. rectangularis* (Nägeli) Gay | *P. simplex* Meyen |
| *C. tetrapedia* (Kirchner) W. & G.S. West | *P. tetras* (Ehr.) Ralfs |
| *Crucigenia* sp. | *Quadricoccus ellipticus* Hortobágyi |
| *Crucigeniella neglecta* (Fott et Ettl) Kom. | *Scenedesmus acuminatus* (Lag.) Chodat |
| *Dictyosphaerium ehrenbergianum* Näg. | *S. acutus* (Meyen) Chodat |
| *D. pulchellum* Wood. | *S. calyptratus* Comas |
| *Didymocystis* sp. | *S. ecornis* (Ralfs) Chodat |
| *Diplochloris decussata* Kors. | *S. intermedius* Chodat |
| *Diplochloris* sp. | *S. linearis* (Ehr.) Chodat |
| *Eutetramorus fottii* (Hindák) Kom. | *S. nanus* Chodat |
| *Franceia amphitricha* (Lag.) Hegewald | *S. obliquus* (T.) Kütz. |
| *F. javanica* (C. Bernard) T. Hortobágyi | *S. obtusus* Meyen |
| *Goelenkinia radiata* Chodat emend. Kors. | *S. opoliensis* P. Richter |
| *Golenkiniopsis parvula* (Voronichin) Kors. | *S. opoliensis* var*. mononensis* Chodat |
| *Kirchneriella aperta* Teil | *S. quadricauda* (Turf.) Bréb. |
| *K. irregularis* (Smith) Kors. | *S. quadricauda* var. *longispina* (Chodat) G.M. Smith |
| *K. microscopica* G. Nyg. | *S. spinosus* Chodat |
| *K. obesa* (GS West) Schmidle | *Schroederia setigera* (Schröd.) Lemm. |
| *K. pseudoaperta* Kom. | *Tetraedrom minimun* (A. Br.) Hansg. |
| *Kirchneriella* sp. | *T. muticum* (A. Braun) Hansg. |
| *Korshikoviella limnetica* (Lemm.) Silva | *T. trigonum* (Näg.) Hansg. |
| *Lagerheimia chodatii* C. Bernard | *Tetrastrum staurogeniaeforme* (Schröd.) Lemm. |
| *L. genevensis* Chodat | **Zygnematales** |
| *L. quadriseta* (Lemm.) G.M. Smith | *C. acutum* (Lyngb.) Bréb. |
| *Lobocystis planctonica* | *C. gracile* Bréb. |
| *Monoraphidium arcuatum* (Kors.) Hindák | *Closterium* sp. |
| *M. circinale* (Nyg.) Nyg. | *Cosmarium quadratulum* (Gay) de Toni |
| *M. contortum* (Thur.) Kom.-Legn. | *C. subtumidum* Nordst. |
| *M. dybowskii* (Woloz.) Hind. & Kom.-Leg. | *C. tinctum* Ralfs |
| *M. griffithii* (Berk.) Kom.-Legn. | *Cosmarium* sp. |
| *M. irregulare* (Smith) Kom.-Legn. | **Volvocales** |
| *M. komarkovae* Nyg. | *Chlamydomonas grovei* G.S. West |
| *M. minutum* (Näg.) Kom.-Leg. | *Chlamydomonas epiphytica* G.M. Smith |
| *M. setiforme* (Nyg.) Kom.-Leg. | **Ulothricales** |
| *M. subclavatum* Nyg. | *Binuclearia* sp. |
| *M. tortile* (W. West & G.S.West) Kom.-Leg. | *Planctonema lauterbornii* Schmidle |
